# Retinal microvascular alterations in patients with active rheumatoid arthritis without cardiovascular risk factors: the potential effects of T cell co-stimulation blockade

**DOI:** 10.3389/fmed.2024.1247024

**Published:** 2024-02-14

**Authors:** Silvia Piantoni, Francesca Regola, Fabrizio Angeli, Alessia Caproli, Annalisa Trovati, Cesare Tomasi, Giulia Chiarini, Claudia Rossini, Claudia Agabiti Rosei, Carolina De Ciuceis, Franco Franceschini, Maria Lorenza Muiesan, Damiano Rizzoni, Paolo Airò

**Affiliations:** ^1^Rheumatology and Clinical immunology Unit, ASST Spedali Civili, Department of Clinical and Experimental Sciences, University of Brescia, Brescia, Italy; ^2^Internal Medicine Unit, ASST Spedali Civili, Department of Clinical and Experimental Sciences, University of Brescia, Brescia, Italy

**Keywords:** rheumatoid arthritis, abatacept, microcirculation, cardiovascular risk, inflammation

## Abstract

**Background:**

The evaluation of microvascular alterations might provide clinically useful information for patients with an increased cardiovascular (CV) risk, such as those with rheumatoid arthritis (RA), being the small artery remodeling the earliest form of target organ damage in primary CV diseases, such as arterial hypertension. The evaluation of retinal arterioles is a non-invasive technique aimed to identify an early microvascular damage, represented by the increase of the wall-to-lumen ratio (WLR) index. Abatacept (ABA), a T-cell co-stimulator blocker, is used to treat RA. A CV protective action was hypothesized for its peculiar mechanism of action in the modulation of T-cells, potentially involved in the pathogenesis of CV comorbidity. The study aimed to non-invasively investigate morphological characteristics of retinal arterioles in a cohort of RA patients treated with ABA.

**Materials and methods:**

Seventeen RA patients [median (25th-75thpercentile) age = 58 (48–64) years, baseline 28-joint Disease Activity Score DAS28-C-reactive protein (DAS28-CRP) = 4.4 (3.9–4.6), body mass index (BMI) = 24.2 (23.4–26) kg/m^2^, rheumatoid factor positive:52.9%, anti-citrullinated peptide autoantibodies positive:76.5%] without known CV risk factors (arterial hypertension, diabetes, hypercholesterolemia, previous CV events, smoking) were evaluated by the adaptive optics imaging system of retinal arterioles before and every 6 months of therapy with ABA (T0, T6 and T12). Office blood pressure evaluation, 24-h ambulatory blood pressure monitoring and tissue-doppler echocardiography were also performed.

**Results:**

A progressive significant reduction of the WLR of retinal arterioles was observed [T0 = 0.28 (0.25–0.30), T6 = 0.27 (0.24–0.31), T12 = 0.23 (0.23–0.26); p T0 vs. T6 = 0.414; p T6 vs. T12 = 0.02; p T0 vs. T12 = 0.009], without significant variations in other parameters. The T0-T12 reduction of WLR was correlated with that of DAS28-CRP (r:0.789; *p* = 0.005). Moreover, a significant reduction of diastolic office blood pressure and a trend for reduction of daily pressure measured by ambulatory monitoring were observed.

**Conclusion:**

In a cohort of RA patients without known CV risk factors, a reduction of retinal microvascular alterations was demonstrated after treatment for 12 months with ABA, in parallel with the reduction of disease activity. These results might suggest the possibility of microvascular abnormalities regression induced by the immune system modulation.

## Introduction

Rheumatoid arthritis (RA) patients have increased cardiovascular (CV) risk as compared with general population (1). Their excess risk for myocardial infarction and ischemic stroke is comparable to that observed in patients with diabetes mellitus (2). RA patients, especially if seropositive, are also at higher risk to develop heart failure (3). This increased risk is not fully explained by traditional risk factors, concomitant therapies, or genetic features, but most likely it can be traced to RA-related systemic inflammatory processes (4). Among other factors, T lymphocytes have been described to have a pivotal role in the pathogenesis of CV comorbidity in RA and in other diseases. CD4 + CD28-negative T cells were first identified in the plaques of patients with unstable angina and expansions of these cells have been reported in a range of CV conditions (5). Moreover, these cells are expanded in RA patients, especially in those with preclinical atherosclerotic changes (6, 7).

Clinicians are forced to use scores which are validated for general population for the screening of CV risk in RA, due to the lack of RA-specific CV event prediction models (8, 9). According to the last updated guidelines, CV risk assessment should be performed every 5 years in patients with low-to-moderate risk, and patients with high risk should receive appropriate treatments (1). Screening for carotid plaques may be useful, if possible, in routine clinical practice (1).

In the evaluation of CV risk in high-risk categories of patients, new techniques can be used to detect early CV alterations in clinical studies (10). Considering that small artery remodeling (i.e., thickened arterial wall) is the earliest form of target organ damage in arterial hypertension, and it has a role in increasing vascular resistance, the evaluation of microvascular alterations might provide clinically useful information extending the determination of traditional Framingham risk factors (11). Assuming that microvascular damage is present with similar characteristics in all vascular districts, retinal vessels might be considered as a window to the heart (12) and even to the brain (13) and can be evaluated by new techniques such as the adaptive optics imaging system (14). A permanent decrease in arteriolar vessel lumen and an increase in arteriolar vessel wall due to, for example, smooth muscle cells proliferation, resulting in a higher wall-to-lumen ratio (WLR), play a key role for microvascular remodeling in chronic disease states (15, 16). Previous studies have shown strong correlations of WLR with age and blood pressure (BP) (17, 18), and higher retinal WLR has been described in older people or in patients with hypertension (17–19).

Recent studies were published on vascular remodeling of the retinal microcirculation in RA, detected with methods which were different from adaptive optics. The reduction of the vessel density was demonstrated in early RA using the optical coherence tomography angiography (20) and an altered retinal microvascular morphology was showed in active RA patients by processing retinal images through a computerized software that allow to calculate the retinal vascular caliber (21, 22).

The role of antirheumatic drugs on CV risk in patient with RA is not yet fully evaluated. Little is known on the effect of abatacept (ABA), a T cell co-stimulation blocker, even if this agent was demonstrated to reduce the number of circulating CD28-negative T cells (23), therefore specifically targeting relevant players in the pathophysiology of CV comorbidity in RA. The aim of our study was therefore to perform an in-depth CV assessment in a cohort of active RA patients before and after therapy with ABA. To better evaluate the role of antirheumatic therapies we focused on a cohort of patients without known traditional CV factors.

### Patients and methods

#### Patients

Seventeen consecutive patients with RA without known traditional CV risk factors (arterial hypertension, diabetes mellitus, hypercholesterolemia, previous CV events, smoking), treated with ABA for at least 6 months (T6), were enrolled in the study between June 2016 and April 2019, but only 11 patients have concluded 12 months follow-up (T12). Ocular diseases, that may interfere with the experimental study with adaptive optics, were excluded due to a preliminary ophthalmologic visit in all the participants. Among 6 patients who did not complete the scheduled follow-up, two were excluded because arterial hypertension was detected at baseline at the 24-h ambulatory BP monitoring (ABPM) evaluation, and anti-hypertensive treatment with calcium channel-blockers was introduced. One patient was switched to another biological disease modifying anti-rheumatic drug (bDMARD) because of a primary inefficacy of ABA. The other three patients did not complete the study because of the SARS/COV-2 pandemic that limited the frequency of face-to-face visits.

The main clinical and demographic characteristics of these patients are shown in Table 1. The study was approved by the Institution Ethics Committee (NP 2276), and patients’ written consent, according to the Declaration of Helsinki, was obtained.

**Table 1:** Baseline demographic, serological and clinical features of 17 total RA patients and the 11 RA patients with a 12-month follow-up.

Features at T0	All the cohort (*n* = 17)	Patients who completed the study (*n* = 11)	Value of *p*
Gender: M/F, *n* (%)	4/13 (23.5/76.5)	3/8 (27.3/72.7)	0.823
Age, years	58 (48–64)	60 (49–64)	0.880
BMI, kg/m^2^	24.2 (23.4–26)	23.4 (21.6–25.6)	0.615
Arterial hypertension, *n* (%)	0	0	n.a.
Diabetes, *n* (%)	0	0	n.a.
Hypercholesterolemia, *n* (%)	0	0	n.a.
Previous CV events, *n* (%)	0	0	n.a.
Smoking, *n* (%)	0	0	n.a.
RF positivity, *n* (%)	9 (52.9)	5 (45.5)	0.698
Anti-CCP positivity, *n* (%)	13 (76.5)	8 (72.7)	0.823
CRP, mg/L	7.1 (2.4–12)	7.5 (4.1–11.5)	0.755
ESR, mm/h	31 (23–34)	29.5 (25.8–33.5)	0.990
DAS28-CRP	4.4 (3.9–4.6)	4.4 (3.8–4.6)	0.999
HAQ	0.5 (0.2–1)	0.2 (0.1–0.7)	0.435
Disease duration, months	72 (18–144)	80 (15–121.5)	0.954
Currently treated with corticosteroid, *n* (%) Daily dose [Prednisone equivalent mg]	14 (82.4) 4.5 (2.8–5)	9 (81.8) 3.6 (2.4–4.7)	0.971 0.518
N of previous csDMARDs	2 (1–2)	1 (1–2)	0.602
Currently treated with csDMARDs, *n* (%)	13 (76.5)	10 (90.9)	0.329
Currently treated with MTX, *n* (%) Weekly dose [mg]	10 (58.8) 15 (10.6–15)	8 (72.7) 13.8 (10–15)	0.562 0.813
Naive to b/tsDMARD, *n* (%)	14 (82.3)	9 (81.8)	0.971
Currently treated with NSAIDs, *n* (%)	8 (47)	6 (54)	0.698

Data are shown as median (25th-75thpercentile). In bold, significant value of *p*s.

BMI, body mass index; RF, rheumatoid factor; Anti-CCP, anti-cyclic citrullinated peptides antibodies; CRP: c-reactive protein; ESR, erythrocyte sedimentation rate; DAS28, 28-joints disease activity score; HAQ, health assessment questionnaire; DMARD, disease modifying anti-rheumatic drugs, conventional synthetic (cs), target synthetic (ts), and biologics (b); MTX, methotrexate; n, number of subjects; n.a., not applicable; NSAIDs, not steroidal anti-inflammatory drugs.

Clinical disease activity and the response to the treatment were evaluated, respectively, with the 28-joints disease activity score based on C-reactive protein (CRP) (DAS28-CRP), and the European Alliance of Association for Rheumatology (EULAR) criteria of Response to the treatment (24).

During the visit at our hospital, each patients did a rheumatologic evaluation at the Rheumatology and Clinical Immunology Unit, and a CV assessment at the Internal Medicine Unit (ASST Spedali Civili University Hospital of Brescia, Italy). Adaptive optics examination, office BP evaluation, 24-h ABPM and tissue doppler echocardiography were part of the CV assessment.

## Methods

### Microcirculation

#### Adaptive optics imaging technique

Adaptive optics apparatus is an improved version of a traditional fundus camera, allowing the investigation of vessels with 20–150 μm of diameter (16). A beam of light enters the eye, and a small amount is reflected out of the eye and into the optical system. Wavefront aberrations in the reflected image are detected by an image sensor and corrected by a deformable mirror. The achieved image resolution is of the order of 1 μm (16). Other details on the technique are described elsewhere (16). The WLR of retinal arterioles is the crucial parameter which was calculated using the formula (arteriole diameter − lumen diameter)/lumen diameter (16). Moreover, the wall thickness and the wall cross-sectional area (WCSA) were also measured.

### Macrocirculation

#### Blood pressure measurements

##### Office BP evaluation and 24-h ambulatory BP monitoring (ABPM)

BP was measured three times by the same physician in all subjects in a sitting position after 10 min at rest, using a sphygmomanometer and taking the disappearance of phase V Korotkoff sounds as diastolic pressure. Hypertension was defined as a sustained increase in BP (systolic BP > 140 mmHg and/or diastolic BP > 90 mmHg) according to the World Health Organization/ International Society of Hypertension guidelines (25, 26).

Twenty-four-hour BP and heart rate were evaluated by non-invasive automatic monitoring (model 90,207; SpaceLabs, Redmond, WA, USA) (23). The procedure was described elsewhere (27). The 24-h BP profiles were used to calculate mean 24-h systolic and diastolic values, mean daytime systolic and diastolic values, mean night-time systolic and diastolic values (27).

### Tissue Doppler echocardiography

The left ventricular (LV) internal dimensions, interventricular septum and posterior wall thickness were measured according to the American Society of Echocardiography’s recommendations (25). Relative wall thickness was calculated, and values ≥0.43 was considered to indicate left ventricular (LV) concentric geometry. The formula of American Society of Echocardiography was used to calculate LV mass and it was indexed by body height to the 2.7 power (LVMI). LV hypertrophy was defined if LVMI was greater than 47 g/m^2.7^ in women or 50 g/m^2.7^ in men (28). Trans mitral flow velocity profile was evaluated by the Doppler technique, with the sample volume placed at the tips of mitral leaflets from the apical four-chamber view, and the peak early (E wave) flow velocity, peak late (A wave) flow velocity, and the E wave deceleration time was measured. LV isovolumic relaxation time (IVRT) was also measured, as previously described (28).

### Statistics

The lack of Gaussian distribution of all the variables were verified by the Kolmogorov–Smirnov test. Data were expressed as the median (25th-75thpercentile). Mann Whitney U test and Wilcoxon-signed rank test were applied to assess variations for quantitative variables, when appropriated. General linear model for repeated measurements was used as a verification test. The correlations between variables were evaluated by Spearman rank correlation test. A *p* value ≤0.05 was considered statistically significant. All analyses will be carried out using the software package GraphPad Prism (version 6) and IBM SPSS.

## Results

### Longitudinal clinical evaluation of the cohort

Patients who completed the 12 month follow up with CV evaluation (*n* = 11) had a progressive improvement of their symptoms during ABA therapy and were progressively treated with lower doses of prednisone (Table 2). Ten of 11 (91%) patients had a moderate response, and one subject had a good response. The results of the improvement of CRP and DAS28-CRP are shown in Table 2.

**Table 2:** Clinical disease activity features of 11 RA patients with 12-month follow-up.

Features (*n* = 11)	T0	T6	T12	Value of *p* T0 vs. T6	Value of *p* T0 vs. T12	Value of *p* T6 vs. T12
CRP, mg/L	7.5 (4.1–11.5)	4.0 (1–7)	5 (2.6–7.4)	0.167	0.320	0.476
DAS28-CRP	4.4 (3.8–4.6)	2.3 (2.1–3.2)	1.8 (1.3–2.3)	**0.002**	**0.001**	**0.025**
Currently treated with corticosteroid, *n* (%) Daily dose [Prednisone equivalent mg]	9 (81) 4.5 (2.8–5)	4 (36) 2.3 (2–3.4)	3 (27) 2.5 (2.3–4.4)	**0.030** **0.003**	**0.010** **0.004**	0.647 >0.999

Data are shown as median (25th-75thpercentile). In bold, significant value of *p*s.

CRP, high sensitivity C-reactive protein; DAS28: 28-joints disease activity score.

### Longitudinal evaluation of microvascular parameters

**Figure 1:**
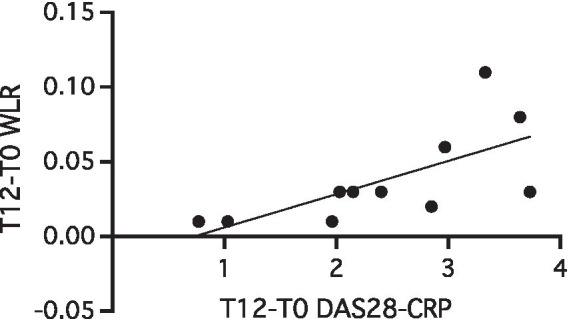
Correlation between T12-T0 variations of WLR and DAS28-CRP in 11 RA patients with a 12-month follow-up (*r*:0.789; *p* = 0.005). CRP, C-Reactive Protein; DAS28, 28-joints disease activity score; WLR, wall-to-lumen ratio; WLR, wall-to-lumen ratio.

As shown in Table 3, five parameters were evaluated through adaptive optics technique on retinal arterioles. Significant reduction of the WLR was observed progressively during time (general linear model, *p* = 0.008). Considering the variation with time of the retinal parameters in correlation with the DAS28-CRP, a significant direct correlation was found between the RA activity index and WLR variations after 12 months of treatment with ABA (*r*:0.789; *p* = 0.005) (Figure 1).

**Table 3:** Retinal arterioles parameters of 11 RA patients with a 12-month follow-up.

Parameters (*n* = 11)	T0	T6	T12	Value of *p* T0 vs. T6	Value of *p* T0 vs. T12	Value of *p* T6 vs. T12
Lumen μm	94.4 (84.1–103.9)	94.8 (84.6–107.7)	99.2 (89.1–109.1)	0.765	0.278	0.278
External diameter μm	125.8 (111.1–131)	122.4 (109.1–134.5)	125.6 (113.9–134.4)	0.898	0.563	0.577
Wall thickness μm	13.2 (12.2–14.4)	13.4 (11.7–14.4)	12.5 (11.6–13)	0.365	0.070	0.175
Wall cross-sectional area μm^2^	4581 (3789–5264)	4563 (3789–5295)	4100 (3899–5146)	0.638	0.365	0.765
Wall to lumen ratio	0.28 (0.25–0.30)	0.27 (0.24–0.31)	0.23 (0.23–0.26)	0.414	**0.009**	**0.002**

Data are shown as median (25th-75thpercentile). In bold, significant value of *p*s.

### Longitudinal evaluation of macrovascular parameters

#### Blood pressure evaluation

Office arterial BP and the 24-h ambulatory BP monitoring were detected in enrolled patients. Longitudinal data are shown in Table 4. Data are presented as the mean values of all the measurements in office, 24-h, day and nighttime. During 12 months of observation, slight variations were observed. A significant decrease of the systolic [T6 vs. T12: 125 (116–133.5) mmHg vs. 116 (110–120) mmHg; *p* = 0.047] and diastolic [T6 vs. T12: 76 (70–76) mmHg vs. 69 (64–71.5) mmHg; *p* = 0.005] office BP was observed (general linear model, *p* = 0.073 and *p* = 0.007, respectively). In the intensive monitoring of the 24-h, the day and night ABPM systolic blood pressure decreased between T0 and T12. value of ps were approaching the level of significancy (*p* = 0.055 and *p* = 0.063, respectively). No correlation was found between blood pressure parameters’ reduction and the decrease of WLR.

**Table 4:** Blood pressure parameters (mmHg) of 11 RA patients with a 12-month follow-up.

Parameter (*n* = 11)	T0	T6	T12	Value of *p* T0 vs. T6	Value of *p* T0 vs. T12	Value of *p* T6 vs. T12
Systolic OBP	120 (115.5–132)	125 (116–133.5)	116 (110–120)	0.449	0.125	**0.047**
Diastolic OBP	75 (72–80)	76 (70–76)	69 (64–71.5)	0.847	**0.014**	**0.005**
24-h ABPM SBP	116 (114–132)	119 (115–122)	113 (105–123)	0.813	0.063	0.672
24-h ABPM DPB	74 (71–79)	72 (71–74)	69.5 (66.3–73.5)	0.500	0.098	0.656
Day ABPM SBP	121 (118–135)	124 (120–125)	112 (110–122)	0.625	0.055	0.313
Day ABPM DBP	78 (76–82)	75 (73–78)	72 (69.5–73)	0.500	0.098	0.156
Night ABPM SBP	109 (105–127)	109 (97–113)	99.5 (95–116)	0.625	0.063	0.313
Night ABPM DBP	65 (59–74)	61 (60–63)	61.5 (58–65.5)	0.375	0.063	0.203

Data are shown as median (25th-75thpercentile). In bold, significant value of *p*s.

OBP, office blood pressure. ABPM, ambulatory blood pressure monitoring; SBP, systolic blood pressure; DBP, diastolic blood pressure.

### Tissue Doppler echocardiography

As shown in Table 5, selected parameters of the echocardiography were evaluated. No significant variations were registered with time. Notably, however, a slight decrease of the left ventricular mass index with time [T0 vs. T6 vs. T12: 31.5 (28.2–35.9) g/m^2.7^ vs. 30.4 (26.8–33.8) g/m^2.7^ vs. 30 (26.5–33.2) g/m^2.7^] was observed.

**Table 5:** Tissue Doppler Echocardiography parameters of 11 RA patients with a 12-month follow-up.

Parameter (*n* = 11)	T0	T6	T12	Value of *p* T0 vs. T6	value of *p* T0 vs. T12	*p*-Value T6 vs. T12
LAD cm	3.3 (3.1–3.6)	3.2 (3.1–3.4)	3.1 (3.0–3.3)	0.512	0.278	0.718
LVM h (g/m^2.7^)	31.5 (28.2–35.9)	30.4 (26.8–33.8)	30.0 (26.5–33.2)	0.275	0.083	0.083
RWT	0.33 (0.29–0.36)	0.30 (0.29–0.35)	0.32 (0.30–0.35)	0.563	1	0.747
Dec E (m/s)	203 (160–235)	199 (185–221)	204 (171–234)	0.447	0.320	0.848
IVRT (m/s)	81 (73.5–100.5)	88 (81–92.5)	80 (72.5–86.5)	0.711	0.449	0.206

Data are shown as median (25th-75thpercentile). In bold, significant value of *p*s.

LAD, left atrial diameter; LVM h, left ventricular mass indexed by body height to the 2.7 power; RWT: relative wall thickness; Dec E, E wave deceleration time; IVRT, left ventricular isovolumic relaxation time.

## Discussion

Our study is the first demonstration of the reduction of microvascular alterations detected by adaptive optics technique in a cohort of patients with active RA treated with ABA. The observed alterations at retinal level in our RA patients may be a consequence of inflammation that affects blood vessels, enhancing precocious mechanisms of endothelial dysfunction which are, at least in part, responsible of the excess of CV diseases in these patients if compared with general population (29). Even though rheumatologists are aware about the presence of a higher CV risk in their patients, one of the objectives of the next years may be to propose a new model of evaluation of this risk in RA, and the methods of detection of early micro and macrovascular modifications could have a place. Retinal vessels’ inspection is a standard procedure for assessing microvascular changes in hypertension or in diabetes and it represents an emerging tool to be used also in other field, like that of autoimmune diseases (20–22).

In our study we enrolled RA patients with an active disease and without any modifiable CV risk factors because we wanted to study the inflammation-related CV risk eliminating potential confounding factors. A new technique of evaluation of the retinal arteriolar morphology was used for the first time in RA patients in our study (14). During the period of observation (one year), we showed a significant reduction of the WLR parameter which is a marker of arteriolar resistance. Interestingly, the reduction of the DAS28-CRP index was directly correlated with that of WLR. This let us to hypothesize a possible effect of the reduction of systemic inflammation due to the treatment in the decrease of arteriolar resistance and vascular swelling. Another hypothesis might be that ABA could have an effect in improving endothelial function thanks to its peculiar mechanism of action on the endothelium.

Notably, some parameters related to the health of the macrovascular system also varied in our patients, in particular a significant reduction of diastolic office BP and a trend for reduction of daily pressure measured by ABPM, decreases that were independent from that of WLR. It should be noted that glucocorticoid frequency of use and dosage were also reduced. In two patients, which were excluded by further analysis, anti-hypertensive therapies were introduced after the first visit because of the detection of high level of arterial pressure at the ABPM evaluation. This suggests the potential utility of the use of ABPM to detect arterial hypertension in an early phase in patients with high CV risk. Furthermore, the involvement in this study might have improved the sense of responsibility of the patients in taking care about their lifestyle. Taken together, these elements might be the main determinants of the improvement of some parameters in our cohort even if they may also represent a major limitation of the study, together with the involvement of middle-aged patients with a moderate disease activity and the lack of a control group treated with other biological treatments. Furthermore, also the progressive reduction of the prednisone daily dose might have had a positive effect on the reduction of the microvascular parameters. All those elements may interfere with the outcome of the study. As expected, considering the relatively short period of observation, no variation was instead shown in echocardiography indexes. The stability of macrovascular parameters during 12 months of ABA therapy was previously demonstrated in a similar cohort of RA patients (30), in contrast with a previous study where a worsening of aortic stiffness was found after 6 months of ABA, probably related to an insufficient decrease of systemic inflammation (31).

Currently, the control of disease activity is the most effective strategy to lower CV risk in RA patients thanks to the reduction of the inflammatory burden (32). According to EULAR recommendations, RA patients should be monitored every 5 years or after major changes in antirheumatic therapy, and lipids monitoring, smoking cessation, regular physical activity, and Mediterranean diet should be advised (1). Furthermore, the lipid increasing effects of certain bDMARDs and some adverse effects of not steroidal anti-inflammatory drugs and corticosteroids should be considered in the management of the disease (1).

Among all the bDMARDs, ABA, a lymphocyte co-stimulation blocker, has a rationale to be efficacious in inducing an improvement of endothelial function (33, 34). This might be an additional effect, strictly related to its mechanism of action that can lead to the reduction of CD28-negative T cells in the bloodstream (5). In fact, several studies support a role of circulating T cells lacking CD28 surface molecule in inducing functional impairment of arterial endothelium, that is currently considered to be the earliest stage of atheroma development, but also in enhancing plaque instability promoting CV disease progression (6). This T cell subpopulation may cause an increase in endothelial oxidative markers and in arterial stiffness, with relevant consequences on left ventricular mass (5). Unlike the common helper T cells, CD4 + CD28-negative subpopulation produces a great amount of TNF-alpha, IFN-gamma, perforin and granzyme B which have cytotoxic activity on endothelial cells (35). In a rat model, chronic administration of a potassium channels blocker prevented the development of unstable atherosclerotic plaques by blocking the release of inflammatory and cytotoxic molecules from CD4 + CD28-negative T cells (36).

A clinical study found that ABA was associated with a 20% reduced risk of CV disease in comparison with TNF-alpha inhibitors, among patients with CV disease history (37). So far, there are data from preclinical studies on atherosclerosis (34) and from large population studies confirming its potential CV benefits (37, 38).

As additional evidence of the importance of T cells co-stimulation blocking in the prevention of CV events, some authors demonstrated that ABA administration in animal models of heart failure reduced the severity of cardiac dysfunction and fibrosis, when compared to non-treated animals, even if it was administered late in the disease course (39). In these experiments, the authors showed that the ABA effect was exerted as a combination of the T cells inhibition, but also of macrophage functions with the induction of signals in B cells, triggering a compensating anti-inflammatory IL-10 expression (39).

Considering this last evidence and the results of our study, a fascinating hypothesis that can be postulated is that, in the future, new possibilities for the treatment of CV risk in our patients could be a reality, in addition to prevention strategies. Immunosuppressants, with ABA as a possible preferred candidate among the others thanks to its peculiar mechanism of action, may be used for the cure of CV complications, at least, in inflammatory diseases.

## Data availability statement

The raw data supporting the conclusions of this article will be made available by the authors, without undue reservation.

## Ethics statement

The studies involving humans were approved by the Brescia ethical committee, P.le Spedali Civili 1, Brescia. The studies were conducted in accordance with the local legislation and institutional requirements. The participants provided their written informed consent to participate in this study.

## Author contributions

SP, PA, DR, and MLM contributed to conception and design of the study. SP, FR, FA, AT, GC, CR, CAR, and CDC conducted the study and organized the database. SP, AC, CAR, and CC performed the statistical analysis. SP, FR, PA, FA, CAR, CC, and FF wrote sections of the manuscript. CT in performing the statistical analysis. All authors contributed to manuscript revision, read, and approved the submitted version.
